# TRX-LOGOS - a graphical tool to demonstrate DNA information content dependent upon backbone dynamics in addition to base sequence

**DOI:** 10.1186/s13029-015-0040-8

**Published:** 2015-09-25

**Authors:** Connor H. Fortin, Katharina V. Schulze, Gregory A. Babbitt

**Affiliations:** Thomas H. Gosnell School of Life Sciences, Rochester Institute of Technology, Rochester, NY 14623 USA; Department of Molecular and Human Genetics, Baylor College of Medicine, Houston, TX 77030 USA

## Abstract

**Background:**

It is now widely-accepted that DNA sequences defining DNA-protein interactions functionally depend upon local biophysical features of DNA backbone that are important in defining sites of binding interaction in the genome (e.g. DNA shape, charge and intrinsic dynamics). However, these physical features of DNA polymer are not directly apparent when analyzing and viewing Shannon information content calculated at single nucleobases in a traditional sequence logo plot. Thus, sequence logos plots are severely limited in that they convey no explicit information regarding the structural dynamics of DNA backbone, a feature often critical to binding specificity.

**Software and implementation:**

We present TRX-LOGOS, an R software package and Perl wrapper code that interfaces the JASPAR database for computational regulatory genomics. TRX-LOGOS extends the traditional sequence logo plot to include Shannon information content calculated with regard to the dinucleotide-based BI-BII conformation shifts in phosphate linkages on the DNA backbone, thereby adding a visual measure of intrinsic DNA flexibility that can be critical for many DNA-protein interactions. TRX-LOGOS is available as an R graphics module offered at both SourceForge and as a download supplement at this journal.

**Results:**

To demonstrate the general utility of TRX logo plots, we first calculated the information content for 416 *Saccharomyces cerevisiae* transcription factor binding sites functionally confirmed in the Yeastract database and matched to previously published yeast genomic alignments. We discovered that flanking regions contain significantly elevated information content at phosphate linkages than can be observed at nucleobases. We also examined broader transcription factor classifications defined by the JASPAR database, and discovered that many general signatures of transcription factor binding are locally more information rich at the level of DNA backbone dynamics than nucleobase sequence. We used TRX-logos in combination with MEGA 6.0 software for molecular evolutionary genetics analysis to visually compare the human Forkhead box/FOX protein evolution to its binding site evolution. We also compared the DNA binding signatures of human TP53 tumor suppressor determined by two different laboratory methods (SELEX and ChIP-seq). Further analysis of the entire yeast genome, center aligned at the start codon, also revealed a distinct sequence-independent 3 bp periodic pattern in information content, present only in coding region, and perhaps indicative of the non-random organization of the genetic code.

**Conclusion:**

TRX-LOGOS is useful in any situation in which important information content in DNA can be better visualized at the positions of phosphate linkages (i.e. dinucleotides) where the dynamic properties of the DNA backbone functions to facilitate DNA-protein interaction.

**Electronic supplementary material:**

The online version of this article (doi:10.1186/s13029-015-0040-8) contains supplementary material, which is available to authorized users.

## Background

For most organisms, DNA is the primary information storing molecule in the cell. Since the discovery of its molecular structure [1–3], it had been generally accepted that information in DNA is exclusively contained within its nucleobase sequence. At about the same time, Claude Shannon formalized the most important generalized mathematical definition of information [4] expanding on Norbert Weiner’s work in probability and thus founding the modern statistical subdiscipline of information theory. The application of information theoretics to DNA sequence did not occur until much later, after the advent of automated forms of DNA sequencing, when Schneider and Stevens [5] utilized Shannon information in the form of the ‘sequence logo’ plot to describe empirically-determined binding sequence preferences of transcription factors.

Historically, studies of transcription factor binding sites (TFBS) have focused primarily upon DNA nucleobase sequence at points of major groove contact, where base sequence is directly readable at the molecular level. Thus, these studies have been largely confined to a single-dimensional bioinformatic perspective. However, recent studies have illuminated the importance of a more biophysical and less sequence-specific second mechanism involved in TF binding, whereby TF’s are drawn near to their cognate sites through a process commonly referred to as “indirect readout” [6–12], or “shape readout” [13]. This form of recognition of local DNA sequence occurs without direct base contacts at the major groove, and is dependent upon local DNA shape, deformability (e.g. an ability to kink) and charge. The ability of any given sequence to bind through indirect readout is also related to the base sequence by its correlation with the local spacing of phosphate groups determining the width of the minor groove [14] as well as the dynamic behavior of phosphate linkages that define the structural dynamics of dinucleotides in aqueous solution [15, 16]. Thus, indirect readout can involve many complex correlated aspects of both DNA’s structure and structural dynamics [17]. DNA shape involves subtle local sequence-dependent variations in minor groove width and subsequent electrostatic charge [14]. The structural dynamics of DNA can be observed in its intrinsic flexibility measured freely in solution as BI-BII conformation shifts in phosphate linkages on the backbone [15] and DNA flexibility has been recently become the subject of both evolutionary and global mapping studies in yeast and other micro-organisms [16, 18–20]. It is also readily observed in measurements of its deformation energy to the nucleosome core [21, 22], other proteins [23], base stacking energy [24], solvent accessibility [25], and pattern of cleavage by nucleases [26] as well. Many of these biophysical aspects involved in indirect readout are generally correlated to organism-specific genomic features influencing gene function [16, 17] and evolution [20, 27–29] including local GC content [20, 30] and purine-pyrimidine states of nucleobases flanking the phosphate linkages (e.g. YR dimers; [23]). Recent studies have even shown that alignments tuned to the DNA shape can even significantly enhance TFBS identification, again furthering the now well-known understanding of the role of indirect readout in TF binding [31, 32].

While traditional sequence logo plots have wide utility and popularity for describing TFBS [5, 33], they cannot display information content most relevant to indirect readout mechanisms (e.g. structural features affecting major and minor grooves as well as backbone conformation, hydration, deformation and flexibility [9]). This information can depend partly upon the DNA backbone’s structural shape and dynamic conformational states of the phosphate linkages defined over very short sequences (i.e. dimer, trimer or tetramer). There is no reason why an information theoretic such as the Shannon information, cannot be calculated using an alphabet representing the structural dynamic state of any given phosphate linkage. The dynamic states of dinucleotide have recently been reported in a meta-analysis of experimental data by Heddi et al. [15]. They have devised a twist, roll and X displacement scale (or TRX scale) that is a simple, nonlinear metric that relates a given length of DNA sequence to its inherent structural dynamics (i.e. intrinsic flexibility), due to BI – BII chemical shifts in phosphate linkage conformation on the DNA backbone while freely in solution in the absence of other DNA binding proteins [15]. The TRX scale represents the percentage of time a phosphate linkage spends in the BII conformation and ranges from 0 to 43 and is positively correlated to GC content of a given dinucleotide. While the TRX metric ignores higher-order aspects of DNA flexibility, it has been well validated as an excellent predictor of DNA-protein interaction, including nucleosome positioning, as most interactions are generally facilitated by the local landscape of DNA flexibility and charge in the genome. Other less recent empirical metrics of DNA flexibility have also been successfully based on lower-order interactions (i.e. trimer [19]) and recent computer simulation studies of tetranucleotide suggest that the influence of DNA structure on dynamics could extend slightly beyond adjacent nucleobases (i.e. dinucleotides) [34, 35]. Dinucleotide-based TRX scores have been shown to correlate well with tetranucleotide-based minor groove widths [16]. Because the TRX scale is a simple dinucleotide-based numerical scale, and thus treats each phosphate linkage position independently, it can also be easily incorporated into the positional graphical framework of the traditional sequence logo plot. Here we offer TRX-LOGOS, an R graphics module that produces a traditional sequence logo plot that includes the addition of vertical bars representing the information content calculated at each phosphate linkage as well. Shannon information on the DNA backbone in calculated at each dinucleotide position using an alphabet of nine different probabilistic phosphate linkage dynamic states of the TRX scale. In addition to the R module, we also offer a Perl wrapper script that allows TRX-LOGOS to interface with output obtained directly from the Computational Regulatory Genomics database JASPAR [36]. The plotting module uses gray-scaled bars at intervening phosphate linkage positions to represent both the information content of the DNA backbone (height of bar) as well as its average intrinsic flexibility (shaded from black = no flexibility (TRX = 0) to white = high flexibility (TRX = 43)). TRX-LOGOS can easily identify where DNA structural dynamics are functionally important in DNA-protein interaction and where local structural dynamics involved in this interaction may contrast the surrounding genomic background that defines the chromatin context of a binding site [27].

To demonstrate the utility of TRX logo plots, we analyzed the information content of both DNA sequence and DNA backbone for 416 TFBS functionally confirmed for *Saccharomyces cerevisiae* in the YEASTRACT database [37] including 25 bp upstream and downstream flanking regions. We then produced TRX-logo plots for 16 broader TF classifications (e.g. leucine zipper, copper fist, homeobox etc.) defined for *S. cerevisiae* by the JASPAR database. We used TRX-LOGOS in combination with a JASPAR search to compare TP53 tumor suppressor binding sites with different sample sizes and different experimental methods for detection of binding. We also used JASPAR and MEGA 6.0 [38] to produce a visual example of TRX logos plots of Forkhead box (FOX) DNA binding sites combined with a neighbor-joining tree representing the evolution of FOX genes in humans. We also used TRX-LOGOS to investigate a sequence-independent signature of coding region backbone dynamics by center-aligning all *S. cerevisiae* genes at the start codon*.*

## Implementation

### Shannon information content calculation

Similarly to the method of Schneider and Stevens [5], the Shannon information content (IC) at each P linkage (measured in bits) is calculated at each position *i* within the set of center aligned sequences representing consensus matches as well as their flanking regions. IC is given in accordance to the following equation:

1 $$ I{C}_i={ \log}_2k-{H}_i $$

where *k* is the number of total possible symbols for each representation. Consequently, when calculating IC for the nucleotide sequence *k* = 4 in order to account for the four possible nucleotides, which can potentially be found at each location. Since there are nine distinct TRX scores (see Table 1 in [15]), k = 9 when analyzing each matched region by its flexibility. *H*_*i*_ in turn is defined as:

2 $$ {H}_i=-{\displaystyle \sum_{s=1}^k{p}_{s,i}}\times \log \left({p}_{s,i}\right) $$

where *p*_*s,i*_ is the relative positional frequency of the symbol *s* appearing among all samples at the given location *i*. Due to the fact that the single nucleotide representation has a smaller maximum IC than the dinucleotide notation (maximum IC is log_2_(4) = 2.0 bits and log_2_(9) = 3.2 bits, respectively, also noting that two of the ten dinucleotide states share the same TRX score), all IC scores for P linkages were normalized to 2.0 bits on the Y axis of the plots (as per standard sequence logos plots based on a 4 letter alphabet).

The plotting module produces a traditional sequence logos plot with additional gray-scaled bars at intervening phosphate linkage positions to represent both the information content of the DNA backbone (height of bar) as well as its average intrinsic flexibility (shaded from black = no flexibility (TRX = 0) to white = high flexibility (TRX = 43)).

### Components of the R graphics module and Supplementary downloadable files

We include three supplementary download files. Additional file 1 includes three folders that include the TRX-logos R module, a Perl script wrapper for analyzing JASPAR database output, and a Perl script for general batch processing of position weight matrix (PWM) data files as well as an introductory README.txt file that introduces the package. The folder with the R module (“TRXlogosRmodule”) includes the following files.

README.txt and DESCRIPTION.txt - describes the implementation and usage

logos.R – main R source code for the graphics program

Working-Test.R – subroutine enforces conditions on input data and supplies warnings if format is not correct

get TRX.R and calcTRX.R – subroutines calculate DNA flexibility on DNA sequences

readPWM.R – subroutine returns position weight matrix regarding identity states of nucleobases

trxPWM.R – subroutine returns position weight matrix regarding dynamic states of phosphate linkage

calcTRXIC.R – subroutine returns Shannon information and average DNA flexibility at each position

The folder “PerlWrapperForJASPAR” contains two files.

trxLogos.init – a file where users enter full path of the R binary and the working directory

trxLogosWrapper.pl – the Perl code script for handling JASPAR output.

The folder “PerlScriptForBatchProcessing” contains only the Perl script BatchTRXlogos.pl. NOTE: full path to R binary and working directory are hard coded at the top of the script and must be modified to match your system.

### Implementation of the R graphics code

For single plotting, an R Shiny version of TRX-LOGOS can be implemented at the following website (http://people.rit.edu/gabsbi/TRXlogos.html). TRX-LOGOS is also available as an R graphics module offered on the journal website (Additional file 1) and SourceForge (http://sourceforge.net/projects/seqlogotrx/files/). A Perl wrapper script (BatchTRXlogos.pl) is also included for batch processing multiple files. This package expands on the seqLogo package from Bioconductor and was built with R version 2.15.2. To set up the package, simply place all R scripts in the same directory. Open the file “logos.R” in your R studio console, and execute the source. This will create a function in your workspace called “logos”. The R script is called as follows:

logos(file, sourcefile, update = TRUE, adjust = TRUE)

file: The location of the sequence file. Sequence files have a very simple format; only the center aligned sequences are necessary. One sequence per line in the file.

source file: The location of the file where the helper scripts and logos.R were saved. This allows the program to find them and load them into your workspace. NOTE: Only needs to be supplied if update is true.

update (default TRUE): Will attempt to install the seqLogo package and update the dependency tree. Will also try and call helper R scripts to load them into the workspace. Not necessary unless the helper scripts are not in your workspace, or you do not have the seqLogo package already. NOTE: If this is true, sourcefile must be supplied, otherwise an error will be thrown.

adjust (default TRUE): After the graphic is produced, the program will enter a small console in which the user can manually adjust some of the graphical parameters (bar start location and distance between bars, or both). The script will create a new graphic with your custom parameters without recalculating, improving the speed on this functionality. The default bar parameters work well with sequences around 20 bp. If you are consistently plotting larger or smaller sequences, or simply unhappy with the default parameters, they can be edited in the Working-Test.R file. Simply change the default values (start and increment only) in the function declaration. You will need to have the update function turned on in order to insert the new source into the “seqLogo” package at least once after changing the parameters of the function. This functionality can be turned off when it is undesired, or if you intend to use this script inside of loop.

### Implementation of the R module using the Perl wrapper script

A Perl wrapper script named trxLogosWrapper.pl is also provided in Additional file 1 and can be run by simply typing the following instruction at the command line.

perl trxLogosWrapper.pl [sequence file name] [filetype] [output file]

For PC, it requires the installation of the free Community Edition of ActivePerl 5.16 or higher and a suitable text editor. We recommend Activestate’s freeware version of the Komodo Editor (Komodo Edit 8.0).

This script reads in a sequence file, and constructs a TRX logo plot with all of the sequences in the file. It handles multiple file types.

[sequence file name] = name of file to be read in. Entire path is needed unless file is in the same folder as this script.

[filetype] specifies what kind of format your sequence file is in.

Recognized values for [fileype]:

fasta = .fasta filetype. Will perform center alignment across entire sequence, so it assumes that any flanking sequences on each side of any consensus motif are of equal length

jaspr = .fasta files downloaded from the JASPR database. In these files, the consensus motif is denoted by capital letters. TRX-LOGOS will perform center alignment to align the consensus motif noted by capital letters.

Warning: any files that have a space in the name must be passed in quotes :“[filename]”.

## Methods

To demonstrate the utility of TRX logo plots in a variety of contexts, we conducted a series of analyses of TFBS ranging from yeast to humans. All of these analyses were connected in some way to the JASPAR database but in several examples also includes comparisons to additional data obtained from other sources. We include a whole genomic analysis of all TFBS in *S. cerevisiae* organized using broad JASPAR classifications*,* a comparison of human TP53 tumor suppressor binding sites determined using different methods of capturing TP53 bound DNA, an evolutionary analysis of Forkhead Box/FOX comparing the evolution of the protein and its binding site signature, and finally, a general comparison of information content of DNA backbone in both noncoding and coding region.

### A general whole-genomic analysis of TFBS in yeast

Previously published sensu stricto yeast alignment files including *S. cerevisiae* sequences were downloaded [39], filtered to exclude genes with masked low complexity regions, poor aligning regions and then parsed, removing gaps, to extract 4222 high quality gene coding regions and upstream non-coding regions. 416 consensus sequences for all functionally confirmed TFBS corresponding to each ORF in the Yeastract database [37] were obtained and matched to all putative binding sites on all of their functionally annotated genes. TRX logo plots were produced for each center aligned consensus ± 7 bp flanking region from its total set of functionally confirmed genes collected over the entire genome. 416 Bonferroni corrected t-tests were conducted on all functionally confirmed consensus sequences comparing the average information content obtained at the nucleobase level to that obtained at the phosphate linkage (i.e. backbone) level (Additional file 2). A similar set of 416 tests were conducted on the 50 bp region flanking the confirmed consensus sites as well. 16 general classifications of transcription factors were obtained from the JASPAR TF database [36]. These classifications include beta beta alpha zinc finger, leucine zipper, fungal zinc cluster, homeobox (helix-turn-helix), Myb (helix-turn-helix), winged helix-turn-helix, copper fist, homeobox, GATA, MADS box, HMG (high mobility group) box, Ig-fold, KilA-N, E2F, Forkhead box and Other. The sequences obtained for the 416 Yeastract consensus sequences were collated according to 16 JASPAR classifications to produce general TRX logo plot signatures for each TF class above. The general signatures of each broad classification were assembled (Additional file 3) and discussed in relation to what is known about the functional role of indirect readout in each class.

### A comparison of human TP53 binding sites collected with differing experimental methodologies

A quick search for TP53 on JASPAR finds two datasets, a large dataset JASPAR ID number MA0106.2 including 1231 binding sites isolated using the current next-generation sequencing methods to isolate and sequence reads (i.e. ChIP-seq) and a small dataset JASPAR ID number MA0106.1 obtained with a much older method previously known as CASTing (cyclical amplification and selection of targets) [40] but is now known as SELEX (systematic evolution of ligands by exponential enrichment. The primary difference between the two methods is that ChIP-seq isolates and reads all genomic DNA fragments that are preferentially bound to the transcription factor, while SELEX relies upon repeated cycles of an *in vitro* selection process that isolates any sequences that preferentially bind the transcription factor from a large starting pool of random sequences. We also analyzed a much smaller ChIP-seq dataset published by Cui et al. [41] for comparison as well. We used our Perl script trxLogosWrapper.pl to automate the production of TRX logo plots for each dataset using the Fasta file downloads from JASPAR.

### An evolutionary analysis of human Forkhead Box (FOX) protein and FOX binding evolution

A comprehensive search was conducted on JASPAR to obtain datasets for a variety of Forkhead Box or FOX family transcription factors, an interrelated group of DNA binding proteins important in early embryonic development and possessed of a unique ability to bind condensed chromatin during cell differentiation. The human JASPAR datasets for FOX binding sites included the following protein /JASPAR IDs (FOXA1/MA0148.3, FOXD1/MA0031.1, FOXF2/MA0030.1, FOXH1/MA0479.1, FOXP1/MA0481.1). DNA sequences for each FOX TF matching the same human protein in JASPAR were obtained by querying the Homologene database at NCBI with MEGA 6.0 software and performing multiple alignments on mRNA transcripts using MEGA’s implementation of the CLUSTAL alignment algorithm. A neighbor joining tree was then produced, upon which the TRX logo plot can be compared to various branch lengths, with the overarching goal of visually comparing human binding site evolution to the protein evolution on the gene tree. TRX logos plots for the ancestral Forkhead protein in yeast (*S cerevisiae MA0296.1*) and fly (*D melanogaster MA0446.1*) are also shown.

### A comparison of Shannon information content of the DNA backbone in coding and non-coding region

To investigate information content on DNA backbone that might be imparted solely by the organization of codon assignments in the genetic code (as hypothesized and supported in [16]), a TRX logo plot was created for all 4222 *S. cerevisiae* genes center aligned to the start codon. Shannon information content is visually compared in both coding and upstream non-coding regions directly flanking the start codon, in the absence of all nucleobase sequence information. It was expected that a 3 bp periodic pattern of elevated Shannon information at phosphate linkages might be observed in coding region but be absent in upstream non-coding region. To further investigate non-randomness of the genetic code regarding DNA flexibility, a TRX logo plot was also generated for all individual codons in the yeast genome (i.e. aligned to codon position) and flanked with random DNA of equal base composition. This effectively eliminated information content due to specific genetic content, but retains information contained in the organization of codon assignments in the genetic code itself. A bootstrapping procedure on 100 random sets of 10 genes each, followed by one-way ANOVA, was performed to determine if information content at phosphate linkages aligned to codon position was significantly higher than the background.

## Results and Discussion

**Fig. 1:**
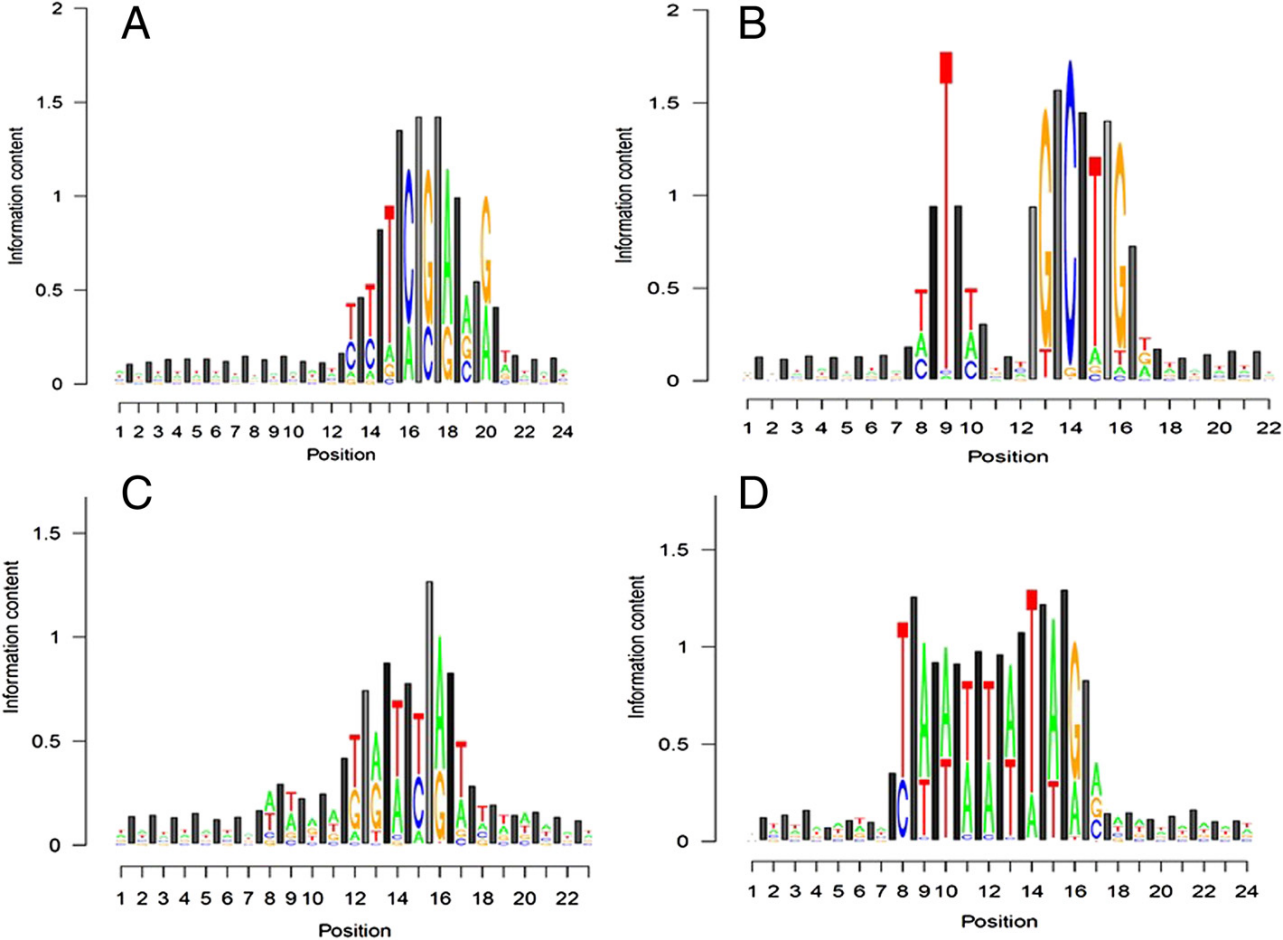
TRX logo plots describing Shannon information content regarding base sequence and intrinsic DNA flexibility at single phosphate linkages in the DNA backbone at functionally confirmed transcription factor binding sites in the *Saccharomyces cerevisiae* genome. Note: lighter shaded bars indicate more flexible conformational states. Functionally confirmed consensus motifs from the Yeastract database were used to search and collect a very large sample of genomic TFBS. These sites were merged to match the 16 general JASPAR database classifications for TFBS. TRX logo plots for four of these merged categories are shown here. These are **a** Ig-fold Rel, **b** Copper Fist, **c** Homeobox and **d** MADS box. All 16 merged TRX-logos category plots are in Additional file 3. Categorizations used are also included in Additional file 3. 7 bp flanking regions were included

**Fig. 2:**
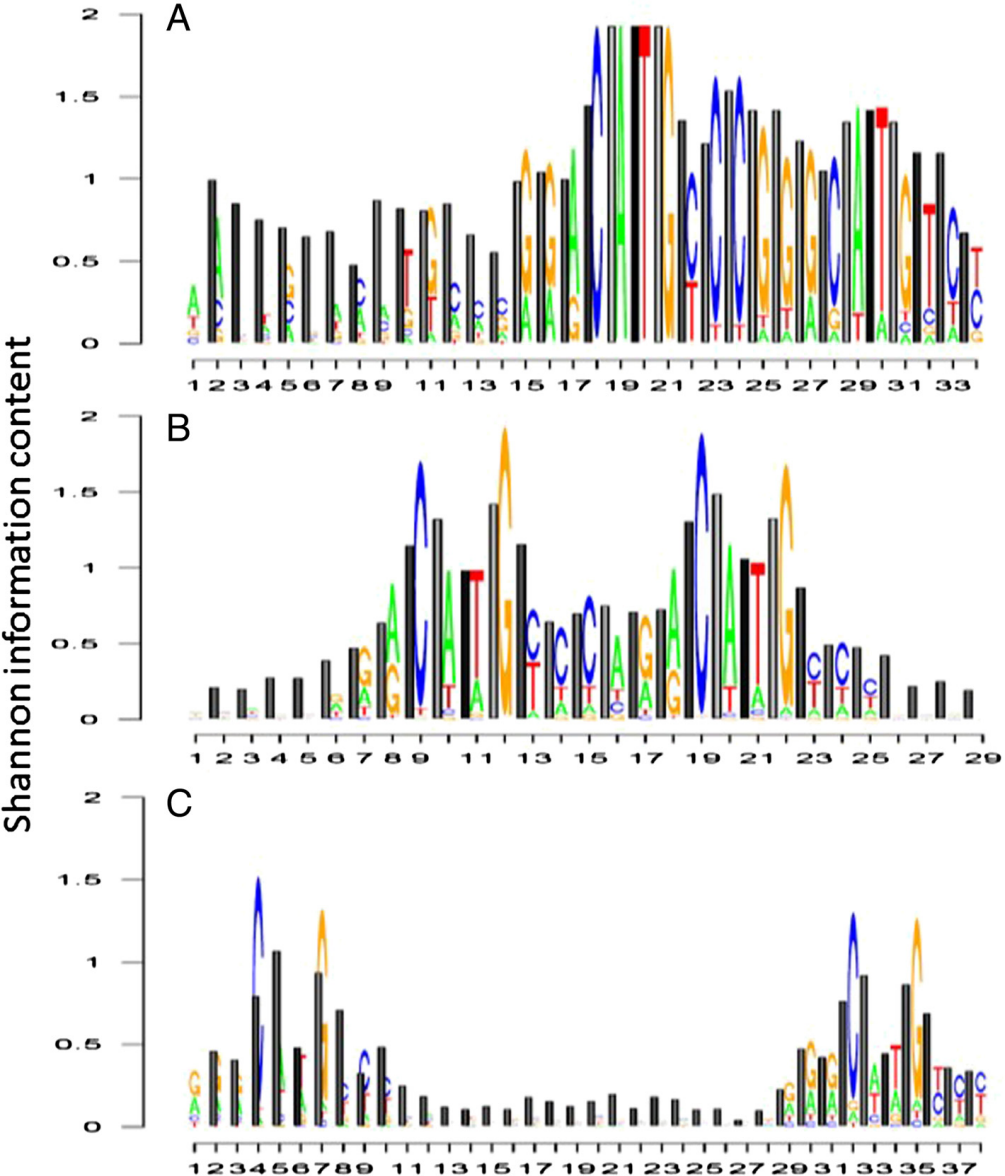
TRX-logos plots describing Shannon information content regarding base sequence and intrinsic DNA flexibility on the DNA backbone for the TP53 tumor suppressor binding site. Lighter shaded bars indicate more flexible conformational states. Shown here are the TP53 binding signatures collected using two molecular biological approaches, (**a**) 17 sites deposited on JASPAR using CASTing or cyclic amplification and selection of targets method of Funk et al. [40] Note: this method is now referred to as SELEX (**b**) 1231 ChIP-seq sites deposited on JASPAR (**c**) 150 sites isolated by ChIP-seq study of Cui et al. [41] (note: sequences were center aligned to each TF bound fragment and flanked to the longest spacer retrieved

**Fig. 3:**
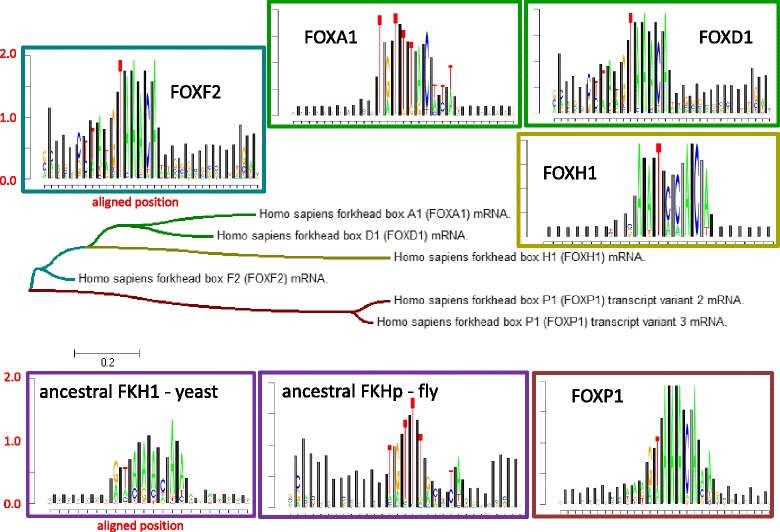
TRX-logos plots comparing Forkhead box binding site evolution to the human FOX protein evolution gene tree. The gene tree was produced using mRNA Clustal alignments downloaded from NCBI and the neighbor-joining algorithm in MEGA 6.0 (Tamura et al. [38]). NCBI identifiers were used to label the tree. TRX-logos plots are color framed to match the branches on the tree. 7 bp flanking regions were included

In the resulting TRX logo plots applied to TFBS (Figs. 1, 2 and 3, Additional file 3), the information content at nucleobases are represented in the standard notation of a traditional sequence logo plot, with stacked colored letters representing the total information and relative frequencies of each base at a given position. The Shannon information content at intervening phosphate linkages is represented by gray-scaled bars normalized to the same y scale as the traditional sequence logo plot. The level of gray in each bar represents the average level of intrinsic DNA flexibility (i.e. average TRX score) ranging from stiff (black; TRX = 0) to flexible (white; TRX = 43).

### A general whole-genomic analysis of TFBS in yeast

To demonstrate the utility of our application in a broad genomic context, we analyzed 416 *Saccharomyces cerevisiae* individual sets of putative matches to TFBS consensus sequences within ORF’s with functionally confirmed regulatory associations defined within the YEASTRACT database [37]. We generated TRX logo plots for each center aligned set of putative matches including upstream and downstream flanking regions. We used t-tests with Bonferroni correction for 416 multiple tests and find that 95 % of all 25 bp flanking regions contain significantly more Shannon information content regarding intrinsic DNA flexibility than can be observed at the single nucleobase level (Additional file 2). This would seem to strongly confirm the importance of the functional evolution of a structurally-defined chromatin context in many TFBS [27, 32]. We further compared TFBS consensus matches in genes known to be targeted by the corresponding TFs to matches in genes, which have not been previously reported to be influenced by the respective TF. Once again applying t-tests with Bonferroni correction for 416 multiple tests, we find that the flanking regions of at least 23 % of TFBS show a significant difference between the two classes. If flanking regions contain more information at phosphate linkage than at nucleobase, it could be because the flexibility of DNA near to the binding site may facilitate recognition.

This leads us to believe that certain groups of TFs may require specific backbone structures to ensure the recognition of the appropriate binding site. We combined YEASTRACT confirmed consensus matches according to 16 broader TF classifications in the JASPAR TF database and generated TRX logo plots for each TF class (Additional file 3). Four of these generalized binding signatures are shown in Fig. 1. TRX-LOGOS software clearly identifies local regions in the binding sequences where the intrinsic flexibility of the DNA greatly contrasts the genomic background and is also much more information-rich when compared to nucleobase sequence. The presence of very light shaded and tall bars in the TFBS signatures for Ig-fold Rel, Copper Fist and Homeobox (Fig. 1a-c) indicate important regions of deformation by kinking (i.e. indirect readout) that are not easily observed in the information content at nucleobases. MADS box (Fig. 1d) does not show this feature and instead demonstrated a simple preference for weak Watson-Crick base pairing without any special local qualities of the DNA backbone. As expected helix-loop-helix and leucine zippers form heterodimers and thus more variability accompanied by a lower information content when generalized across JASPAR classifications (Additional file 3).

### A comparison of human TP53 binding sites collected with differing experimental methodologies

Our TRX logo plots comparing two radically different methods of capturing binding sites for the TP53 tumor suppressor protein (Fig. 2a and b) appear quite different when only the information content at nucleobases are considered (i.e. traditional sequence logo). However, both TRX logo plots demonstrate a clear pattern of alternating kinking and narrow minor groove (which strongly correlates to TRX [16]) across the two major CATG motifs separated by a variable spacer. This is less apparent in the smaller dataset provided by Cui (Fig. 2c; [41]). In general, the YpR steps (i.e. (CpA/TpG, TpA and CpG) tend to kink or hinge due to partial loss of base stacking at the single step indicate by the phosphate linkage. Alternatively, the ApT and ApA/TpT steps exhibit negative roll and the bifurcated H bonds of A:T base pairs tends to enhance propeller twisting and narrowing of minor groove [13]. Very recently, it was discovered that the reduction of the probability of A in the TP53′s CATG motif is strongly associated to reduced specificity of binding as well as increased cooperativity, where deformation of DNA is utilized to achieve the binding of multiple proteins in a larger nucleoprotein complex (Figure 2E in [42]). In our TRX logo plots, we can clearly see that because the ‘A’ in CATG is critically important in defining a very flexible CpA dinucleotide as well as a very stiff ApT dinucleotide, the lost binding specificity when A is removed is clearly a function of properties related to indirect readout. This variability in biophysical characteristics of the CATG motif may help explain much of the pronounced promiscuity of TP53 binding in the genome.

### An evolutionary analysis of human Forkhead Box (FOX) protein and FOX binding evolution

The FOX protein family consists of winged helix-turn-helix motif, where an alpha helix contacts the DNA major groove and an adjacent beta sheet ‘wing’ sits over the minor groove [13]. The involvement of the minor groove naturally led us to speculate a possible role of indirect readout in differentiating many possible aspects governing the specific binding of different members of this group of transcription factors. Indeed, in most FOX binding sites, we clearly observe the role of a CpA hinge following a relatively stiff poly A region when compared to the genomic background. The fly FKH and human FOXA1 data exhibit a similar reverse complemented pattern with a TpG hinge preceding a stiff poly T region. The neighbor-joining tree shows that human FOXH1 and FOXP1 proteins have undergone significantly more evolution (i.e. longer branch lengths) that the other human FOX proteins. Whereas the FOXP1 binding site appears not to have changed much with respect to its binding signature, the FOXH1 protein appears to have replaced its stiff region with a DNA backbone that is much more flexible than the genomic background. Thus, the TRX logo plot is useful in identifying potentially meaningful changes in binding when incorporated into a molecular evolutionary framework.

### A comparison of Shannon information content of the DNA backbone in coding and non-coding region

**Fig. 4:**
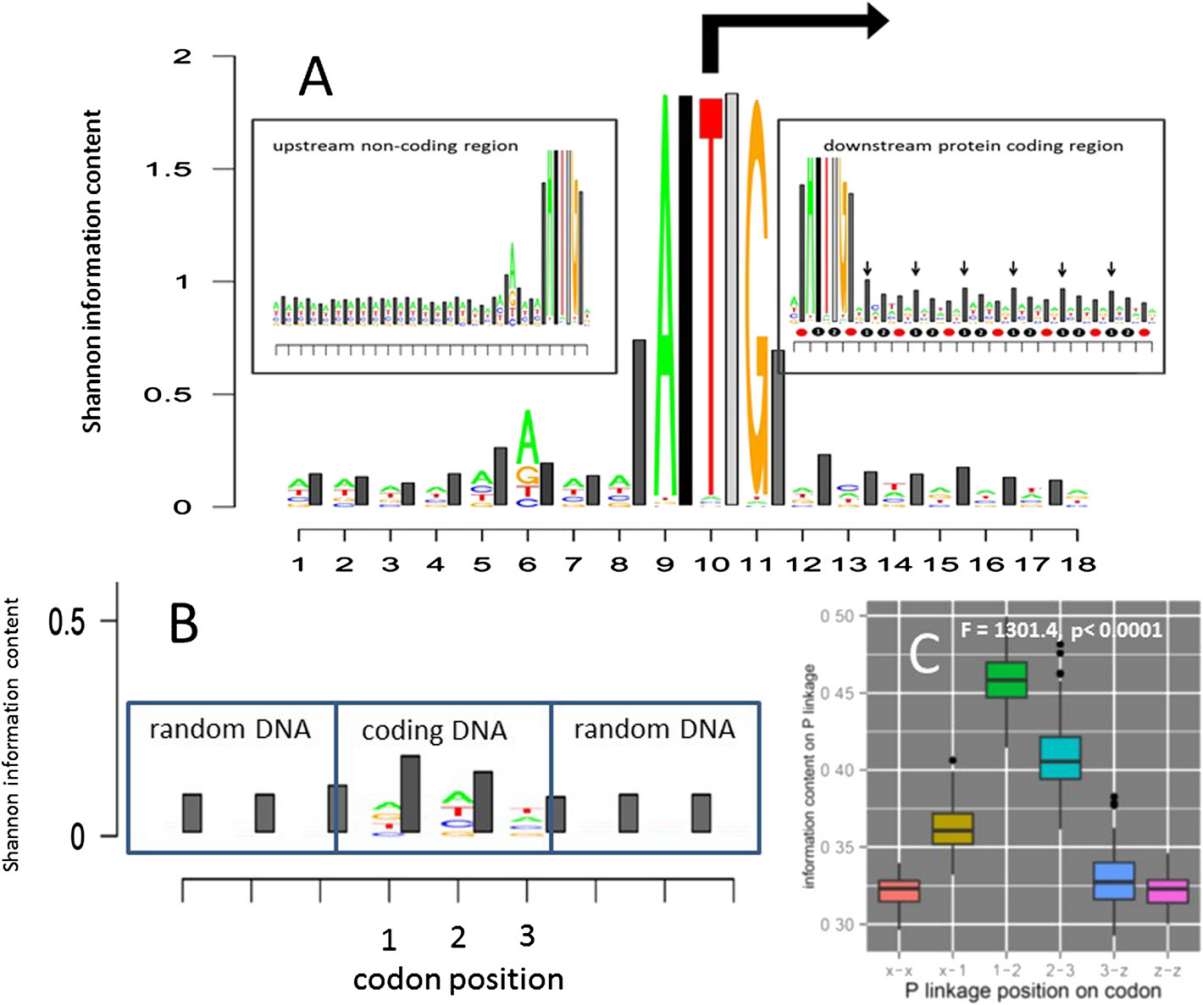
TRX-logos plots comparing non-coding and coding regions for all 4222 *S. cerevisiae* genes in Kellis et al. [39]. **a** The comparison of all genes relative to the start codon indicates a genome-wide persistence in information content at phosphate linkages in coding region in the absence of single site sequence similarity (downward vertical arrows in the right-hand inset mark a 3 bp periodicity). This persistent information does not appear in non-coding regions where bar and letter heights are quite similar. **b** Comparison of information content across all codons compared to flanking triplets of randomly generated DNA also shows a persistent trend where phosphate linkages internal to codons contain slightly more information than random DNA. The phosphate linkages external to codons (i.e. joining adjacent codons) are no more informative than random DNA. **c** Comparison of information content across all codons as in (**b**) but using bootstrap ANOVA for 100 random sets of 10 genes each

A TRX logo plot computed for 4222 ORF’s center aligned on the translation start site demonstrated a pronounced and persistently higher Shannon information content at phosphate linkages in coding region (i.e. all TRX bars are higher than logos lettering) even in the absence of any sequence similarity across sites other than the start codon (Fig. 4a). This persistent and increased level of information content at phosphate linkages shows a clear and marked 3 bp periodicity, with slightly more information occurring at the first internal phosphate linkage of the codon (i.e. between the first and second base position). A comparison of Shannon information of the two internal phosphate linkages to the two external phosphate linkages (i.e. linkages joining adjacent codons) indicates that internal phosphate linkage states of codons contain slightly more information than external linkages or random DNA, even when amino acid sequence is random. As this phosphate linkage connects the two nucleobases that are most instrumental in defining codon assignments to amino acids, its supports our previous conjectures that the genetic code may be optimized with respect to the flexibility of DNA [16, 20]. This persistence in Shannon information at phosphate linkages is not observable in non-coding regions upstream of translation start sites (Fig. 4a inset) and persists well beyond the boundary of Fig. 4a. Average mean Shannon information for each phosphate linkages bridging each codon position across all coding regions in the genome is x-1 position = 0.356 bits, 1–2 position = 0.470 bits, 2–3 position = 0.408 bits and 3-z position = 0.313 (Fig. 4b-c) and are significantly higher than background when bootstrap ANOVA was applied, excepting in the P linkage following the 3^rd^ codon position (F = 1301.4, r <0.0001; Fig. 4c Note: x and z refer to positions of bases in adjoining codons). Thus, the TRX-LOGOS graphic tool would seem to reveal a general property of DNA structural dynamics as it might relate to the nonrandom organization of the genetic code. This signature of the code remains even after the Shannon information content on DNA is computed across thousands of yeast genes aligned to the translation start site, and thereby in the absence of any sequence specific information.

## Conclusion

Our TRX-LOGOS software clearly demonstrates many situations in which important information content in DNA can best be visualized, not at the positions of nucleobases, but rather at the positions of phosphate linkages where the dynamic state of the DNA backbone is functional in maintaining many types of DNA-protein interaction. Our website, open source R package, and supporting scripts allowing interfacing with JASPAR, expands upon the popular sequence logos plotting software already available in the bioconductor software suite and is simple and easy to use. TRX-LOGOS provides researchers with a simple and intuitive way of visualizing information regarding DNA backbone dynamics in an already well-established framework of information theoretics.

## Availability and requirements

- **Project name:** TRX-LOGOS
- **Project home page:**http://people.rit.edu/gabsbi/TRXlogos.html and http://sourceforge.net/projects/seqlogotrx/files/
- **Operating system(s):** Platform independent
- **Programming language:** R
- **Other requirements:** bioconductor module
- **License:** GNU
- **Any restrictions to use by non-academics:** none

## Additional files

Additional file 1:
**The TRX-LOGOS R module download complete with Perl wrapper scripts for batch processing and JASPAR query.  Supporting files and documentation are also included.** (ZIP 14 kb) Additional file 2:
**MS Excel spreadsheets containing 416 Bonferroni corrected t-tests conducted on all functionally confirmed consensus sequences in YEASTRACT database comparing the average information content obtained at the nucleobase level to that obtained at the phosphate linkage (i.e. backbone) level. An additional set of 416 tests were conducted on the 50 bp region flanking the confirmed consensus sites is included as well.** (ZIP 13 kb) Additional file 3:
**TRX logo plots for general signatures of 16 transcription factor binding sites assembled from 416 Yeastract consensus sequences and collated according to JASPAR classification.** (ZIP 1722 kb)

## Abbreviations

DNA: Deoxyribonucleic acid
TFBS: Transcription factor binding site
bp: Base pairs
