# Supplementary figures and images for: TRX-LOGOS - a graphical tool to demonstrate DNA information content dependent upon backbone dynamics in addition to base sequence
Source: Source Code Biol Med. 2015 Sep 25;10:10. doi: 10.1186/s13029-015-0040-8 (PMC4583169; doi:10.1186/s13029-015-0040-8)

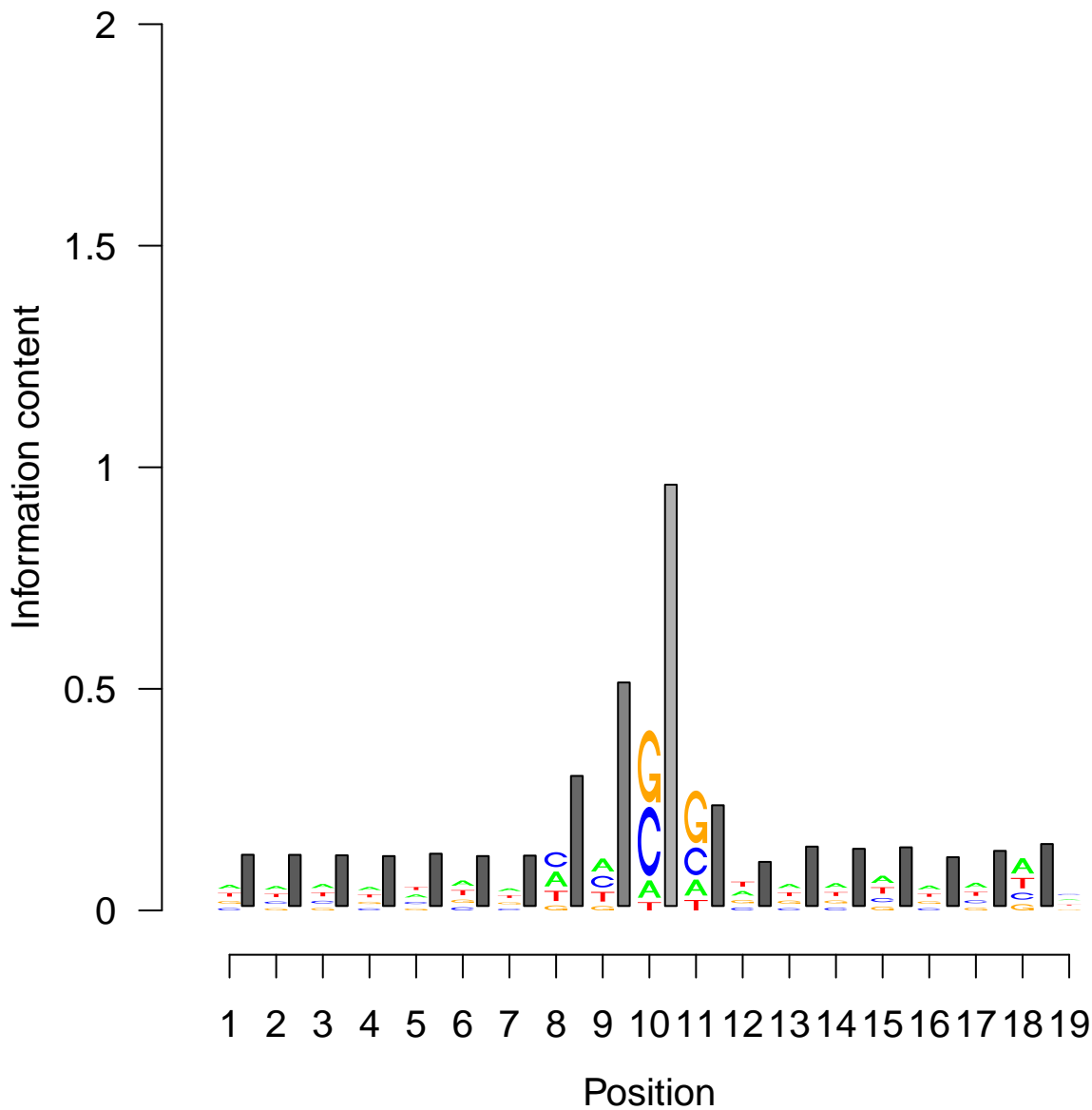

Supplement: Additional file 3: — TRX logo plots for general signatures of 16 transcription factor binding sites assembled from 416 Yeastract consensus sequences and collated according to JASPAR classification. (ZIP 1722 kb) [file 13029_2015_40_MOESM3_ESM.zip › SuppFileC/BetaBetaAlpha-zinc_finger.txt.txt.pdf]

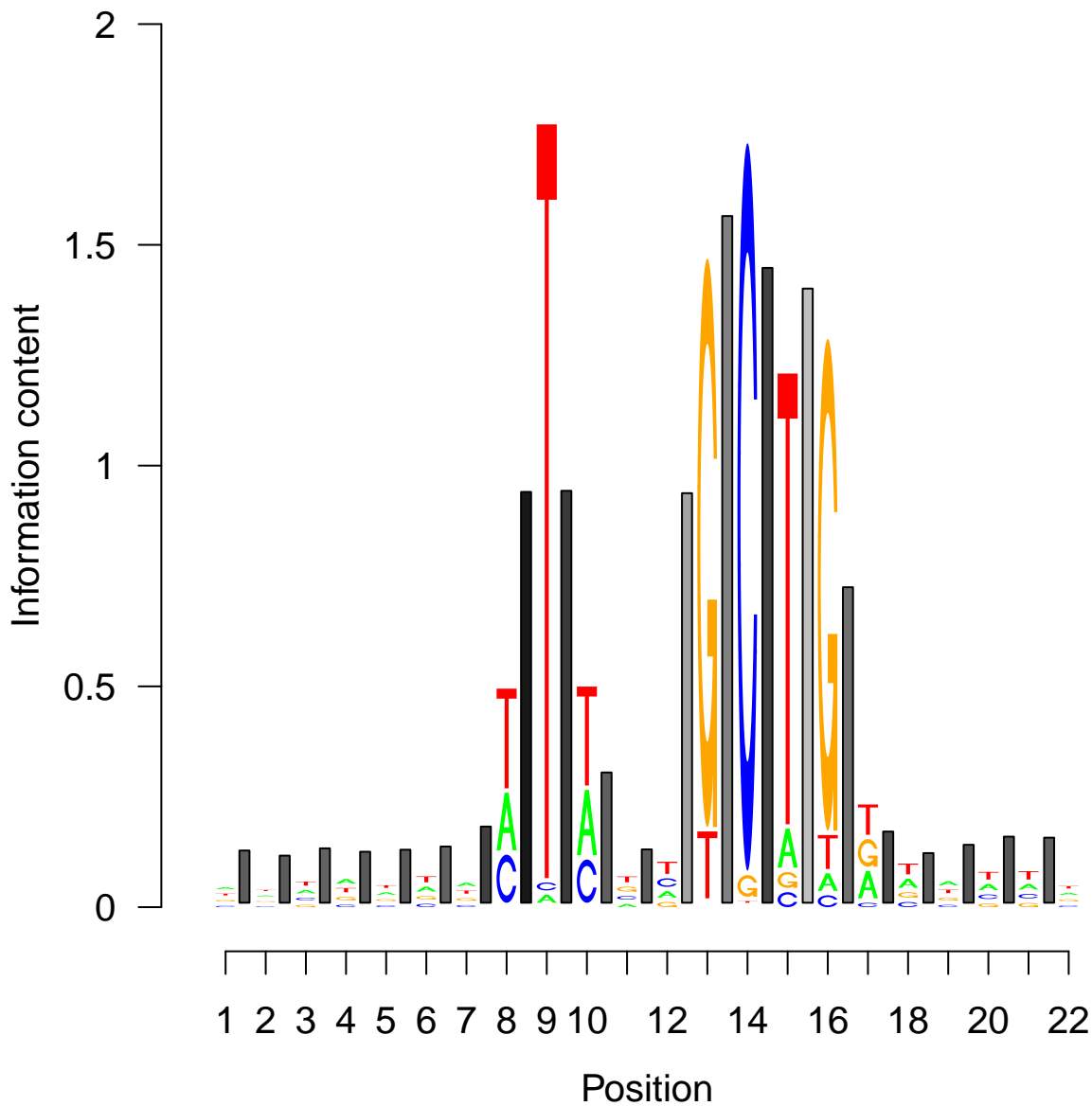

Supplement: Additional file 3: — TRX logo plots for general signatures of 16 transcription factor binding sites assembled from 416 Yeastract consensus sequences and collated according to JASPAR classification. (ZIP 1722 kb) [file 13029_2015_40_MOESM3_ESM.zip › SuppFileC/Copper_fist.txt.txt.pdf]

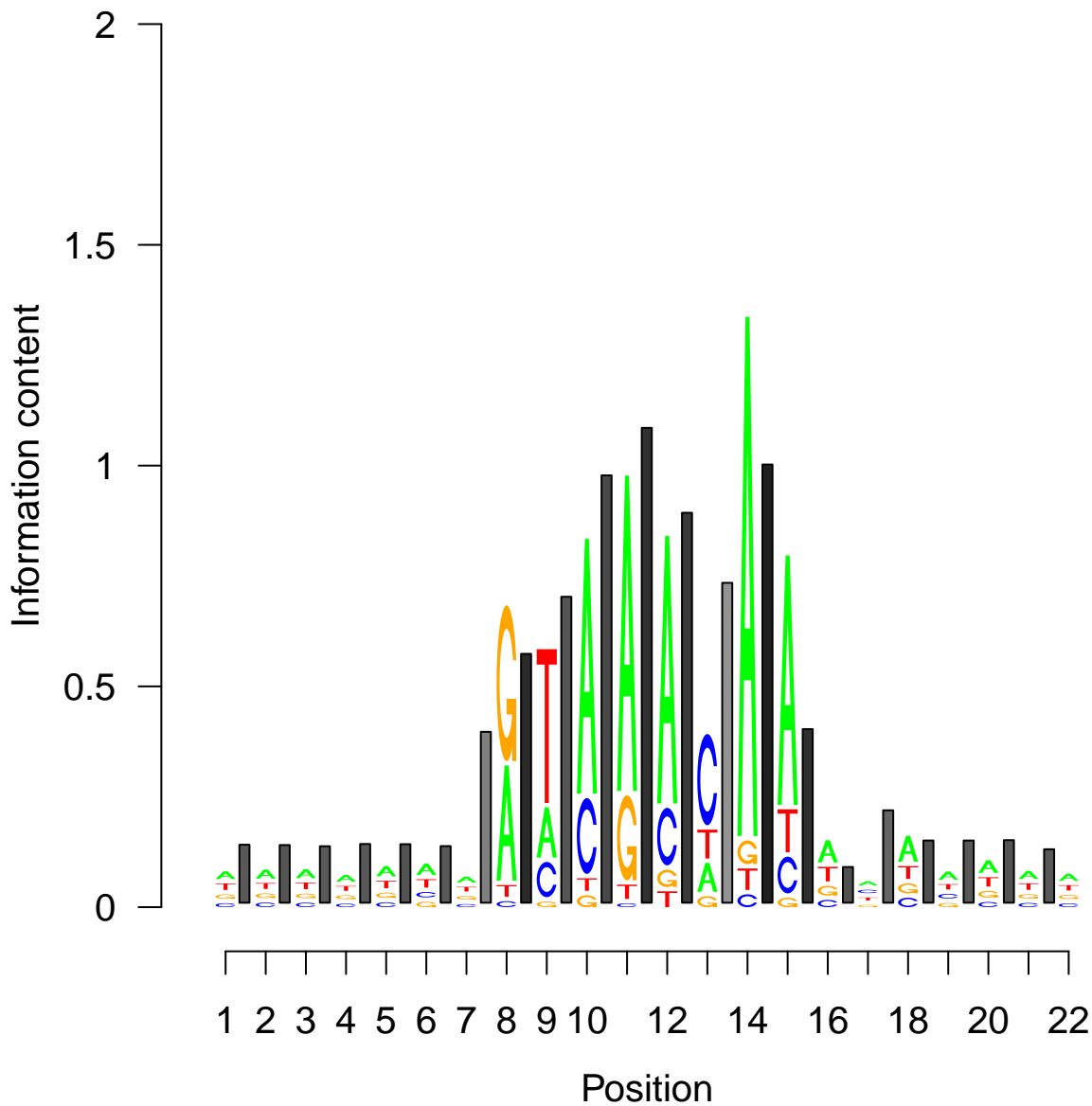

Supplement: Additional file 3: — TRX logo plots for general signatures of 16 transcription factor binding sites assembled from 416 Yeastract consensus sequences and collated according to JASPAR classification. (ZIP 1722 kb) [file 13029_2015_40_MOESM3_ESM.zip › SuppFileC/Forkhead.txt.txt.pdf]

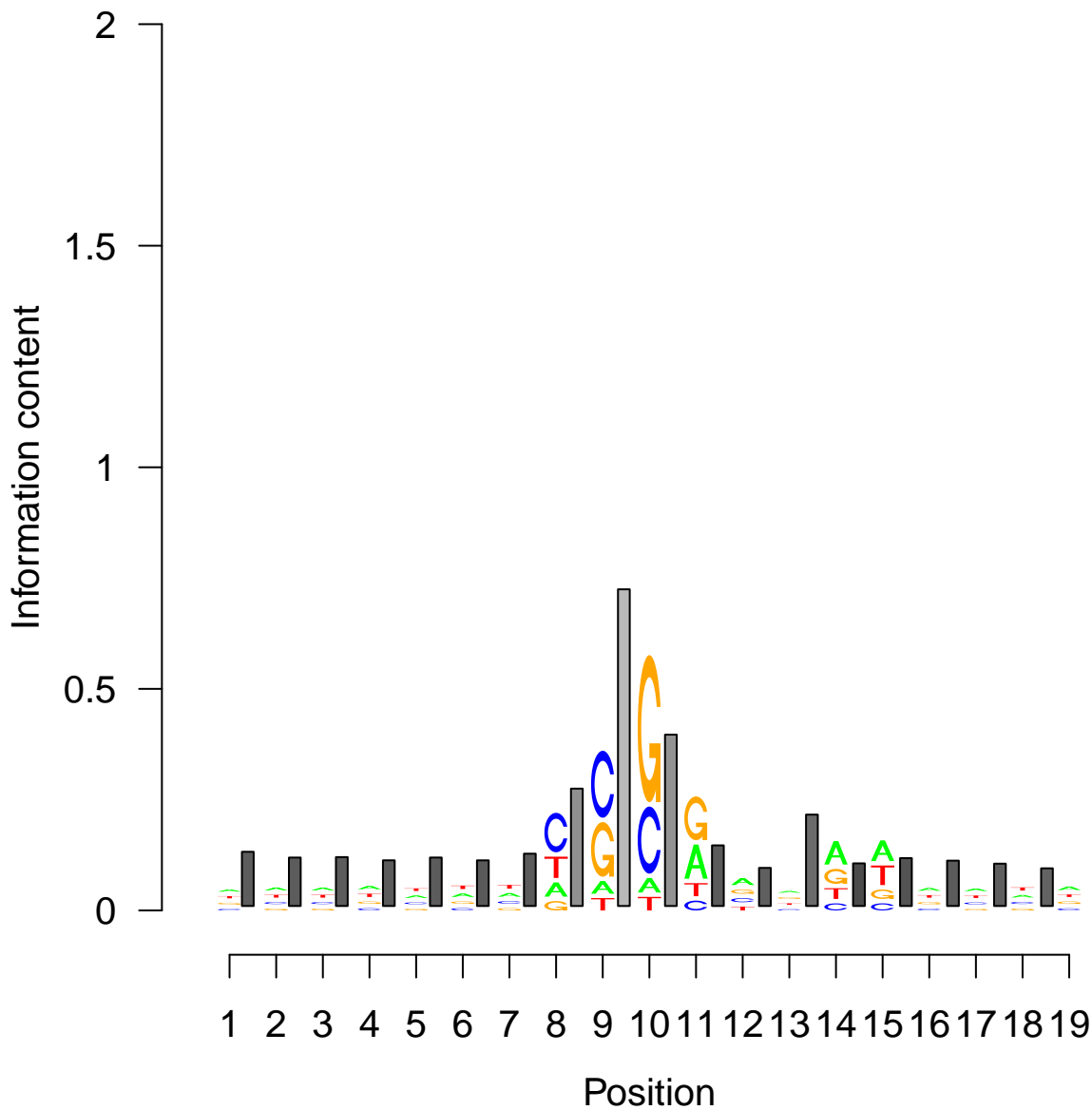

Supplement: Additional file 3: — TRX logo plots for general signatures of 16 transcription factor binding sites assembled from 416 Yeastract consensus sequences and collated according to JASPAR classification. (ZIP 1722 kb) [file 13029_2015_40_MOESM3_ESM.zip › SuppFileC/Fungal_Zn_cluster.txt.txt.pdf]

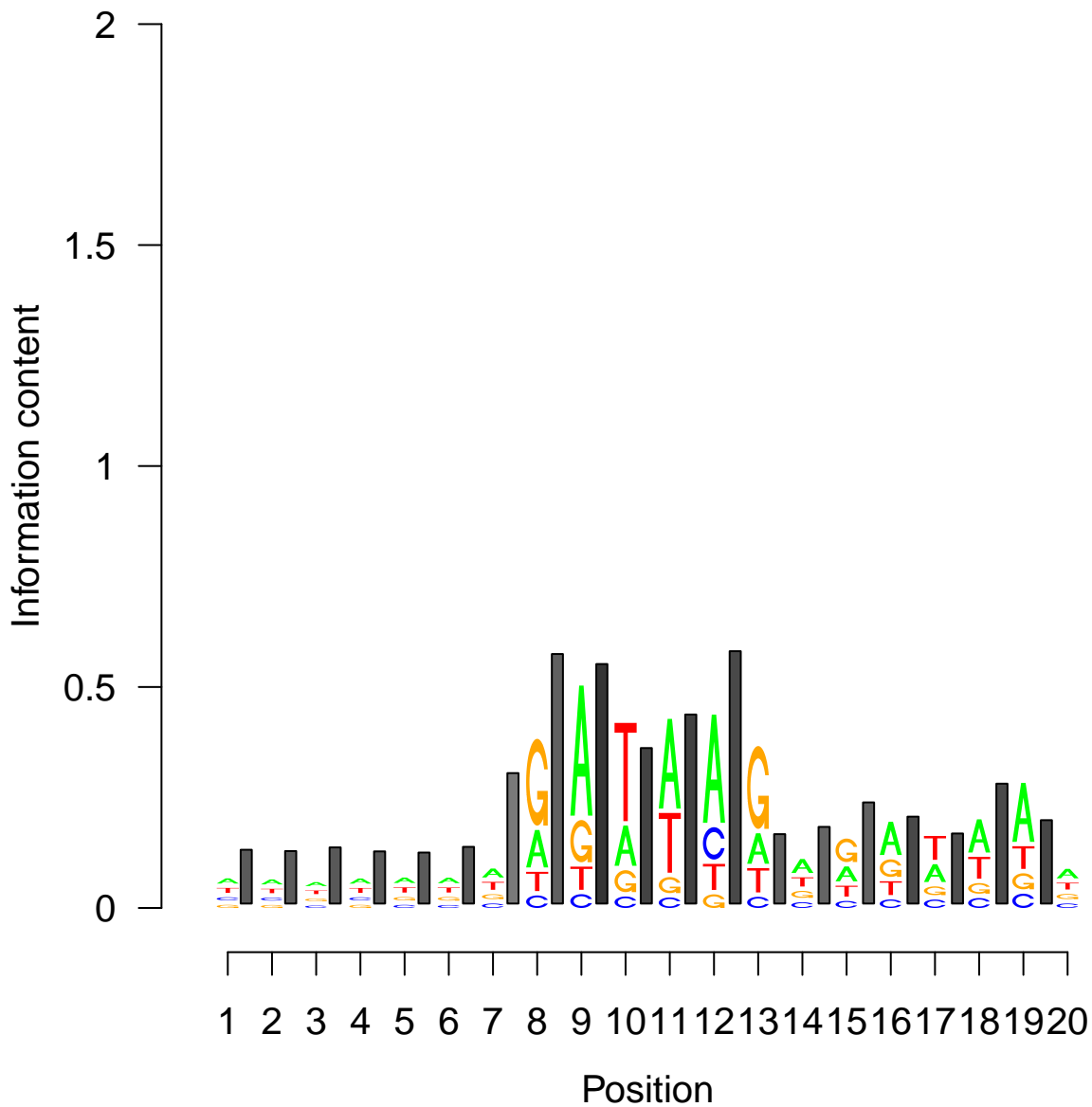

Supplement: Additional file 3: — TRX logo plots for general signatures of 16 transcription factor binding sites assembled from 416 Yeastract consensus sequences and collated according to JASPAR classification. (ZIP 1722 kb) [file 13029_2015_40_MOESM3_ESM.zip › SuppFileC/GATA.txt.txt.pdf]

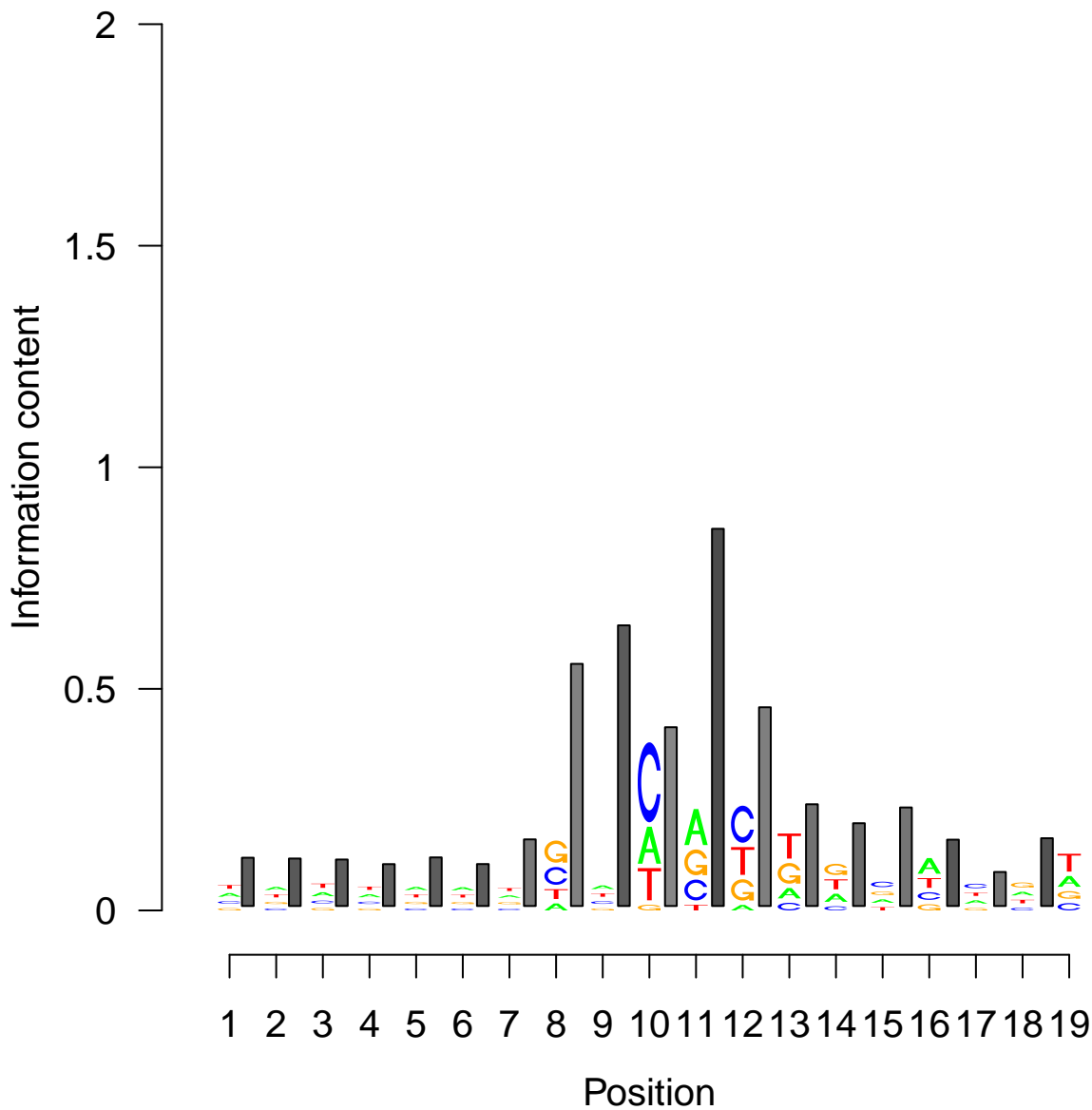

Supplement: Additional file 3: — TRX logo plots for general signatures of 16 transcription factor binding sites assembled from 416 Yeastract consensus sequences and collated according to JASPAR classification. (ZIP 1722 kb) [file 13029_2015_40_MOESM3_ESM.zip › SuppFileC/Helix-Loop-Helix.txt.txt.pdf]

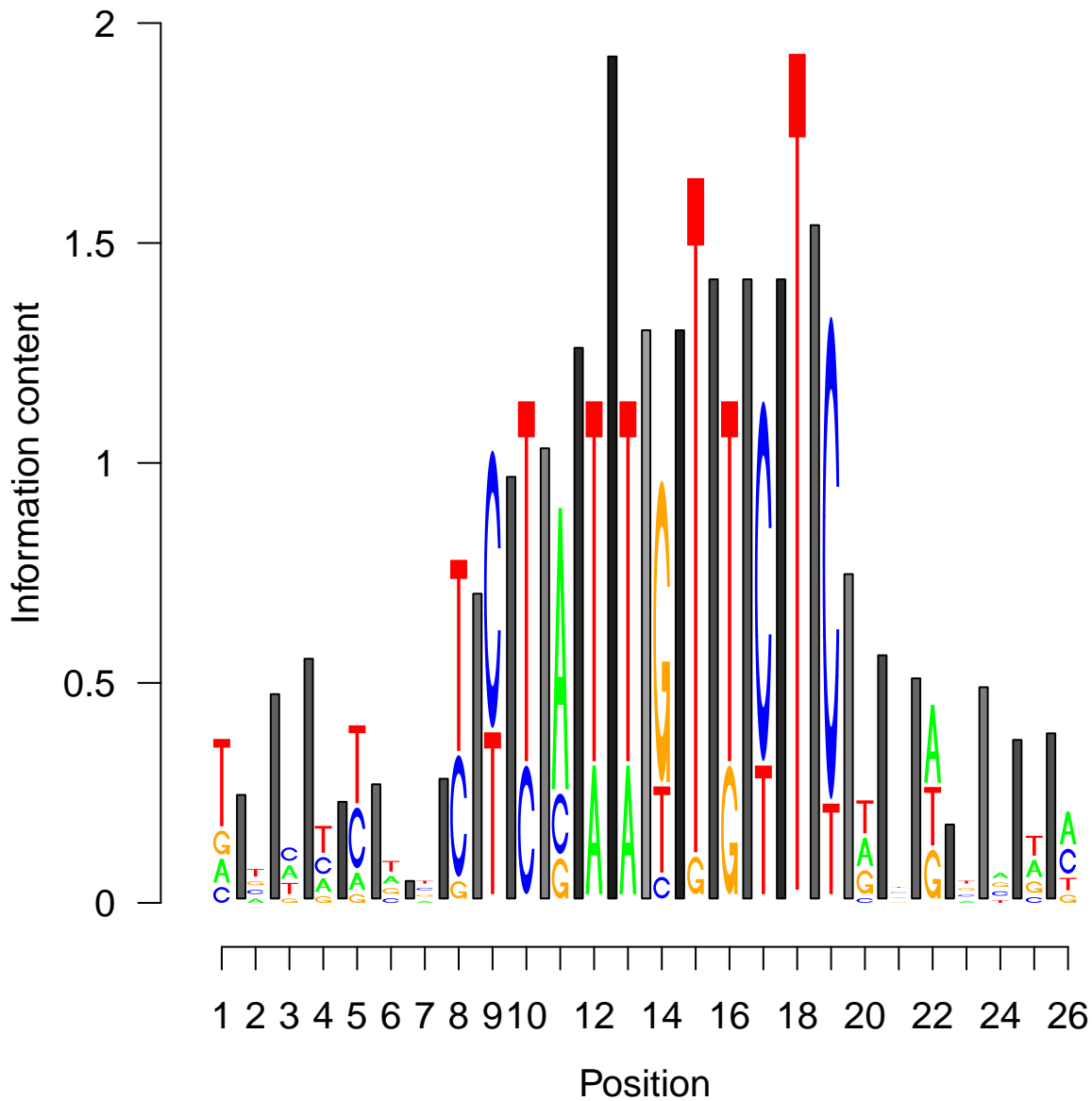

Supplement: Additional file 3: — TRX logo plots for general signatures of 16 transcription factor binding sites assembled from 416 Yeastract consensus sequences and collated according to JASPAR classification. (ZIP 1722 kb) [file 13029_2015_40_MOESM3_ESM.zip › SuppFileC/High_Mobility_Group_box_(HMG).txt.txt.pdf]

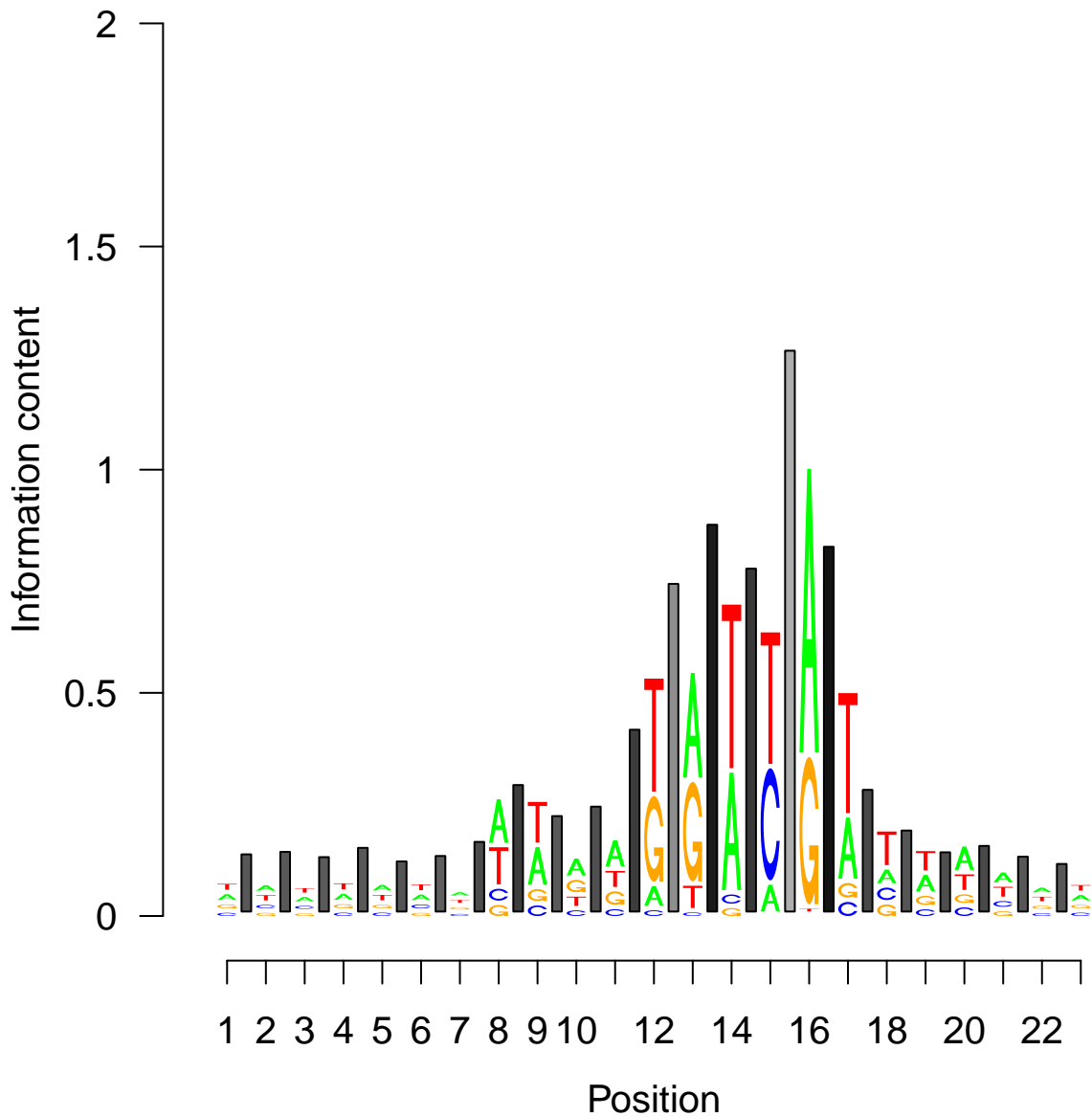

Supplement: Additional file 3: — TRX logo plots for general signatures of 16 transcription factor binding sites assembled from 416 Yeastract consensus sequences and collated according to JASPAR classification. (ZIP 1722 kb) [file 13029_2015_40_MOESM3_ESM.zip › SuppFileC/Homeo.txt.txt.pdf]

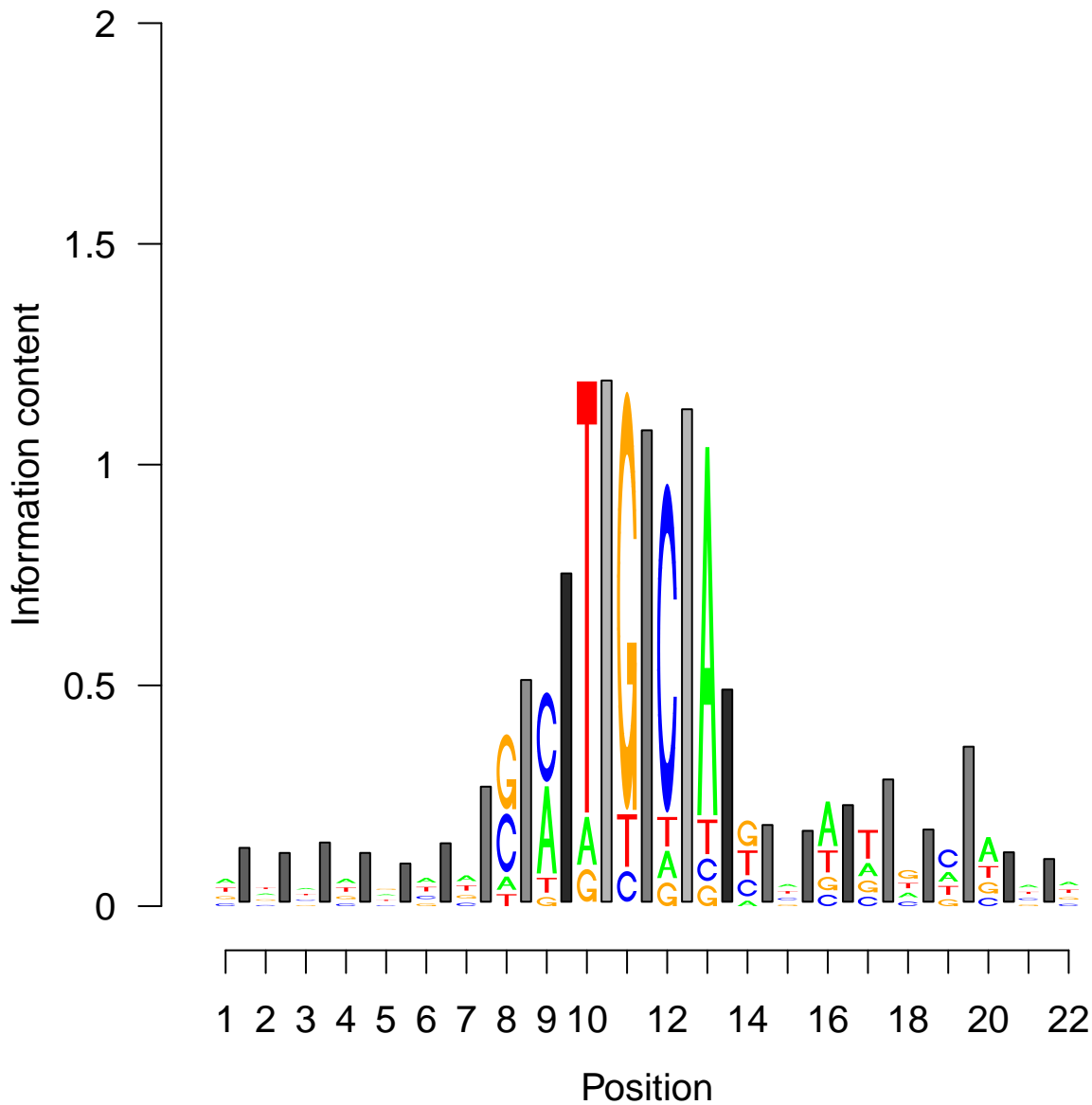

Supplement: Additional file 3: — TRX logo plots for general signatures of 16 transcription factor binding sites assembled from 416 Yeastract consensus sequences and collated according to JASPAR classification. (ZIP 1722 kb) [file 13029_2015_40_MOESM3_ESM.zip › SuppFileC/KilA-N.txt.txt.pdf]

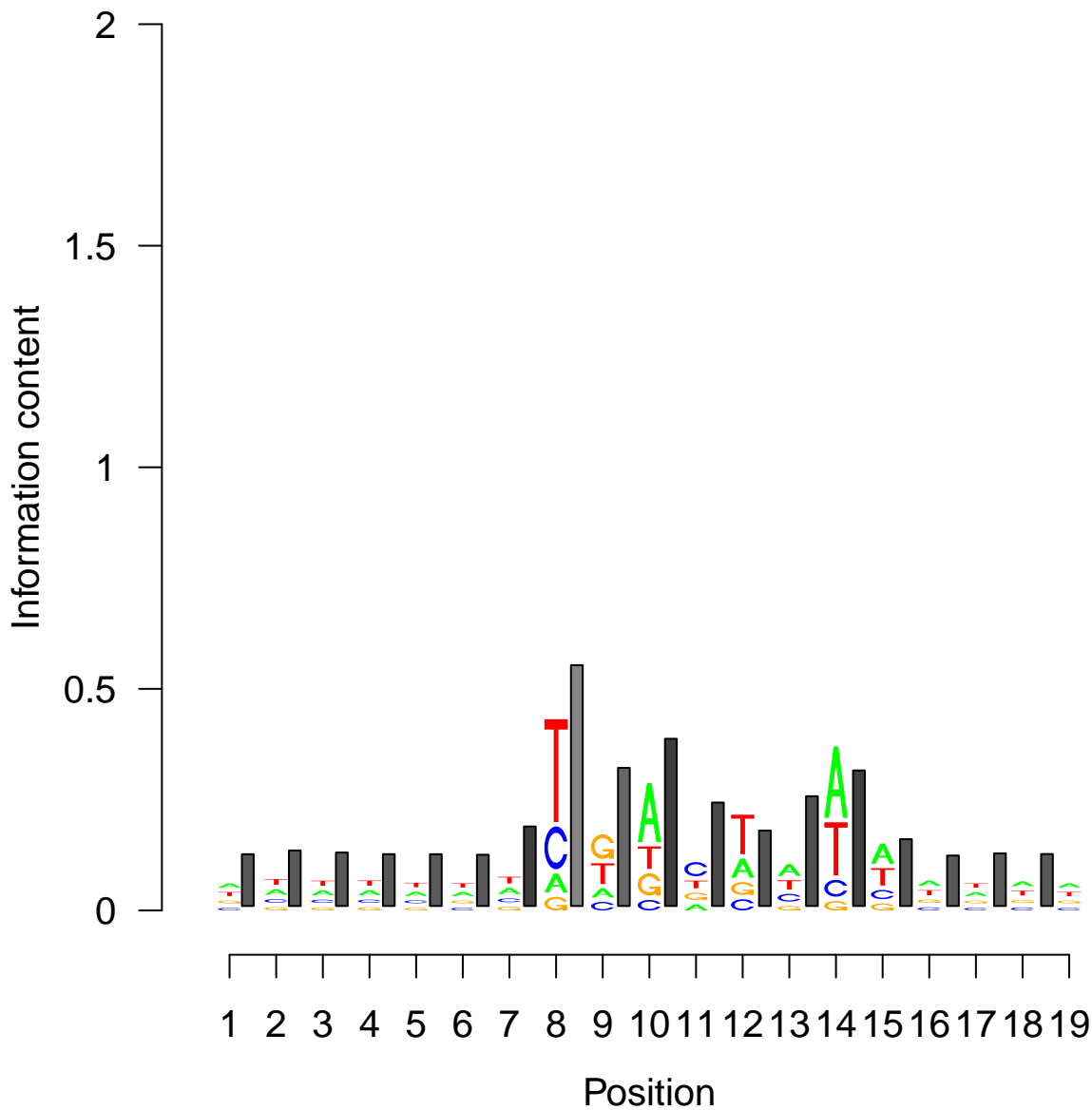

Supplement: Additional file 3: — TRX logo plots for general signatures of 16 transcription factor binding sites assembled from 416 Yeastract consensus sequences and collated according to JASPAR classification. (ZIP 1722 kb) [file 13029_2015_40_MOESM3_ESM.zip › SuppFileC/Leucine_Zipper.txt.txt.pdf]

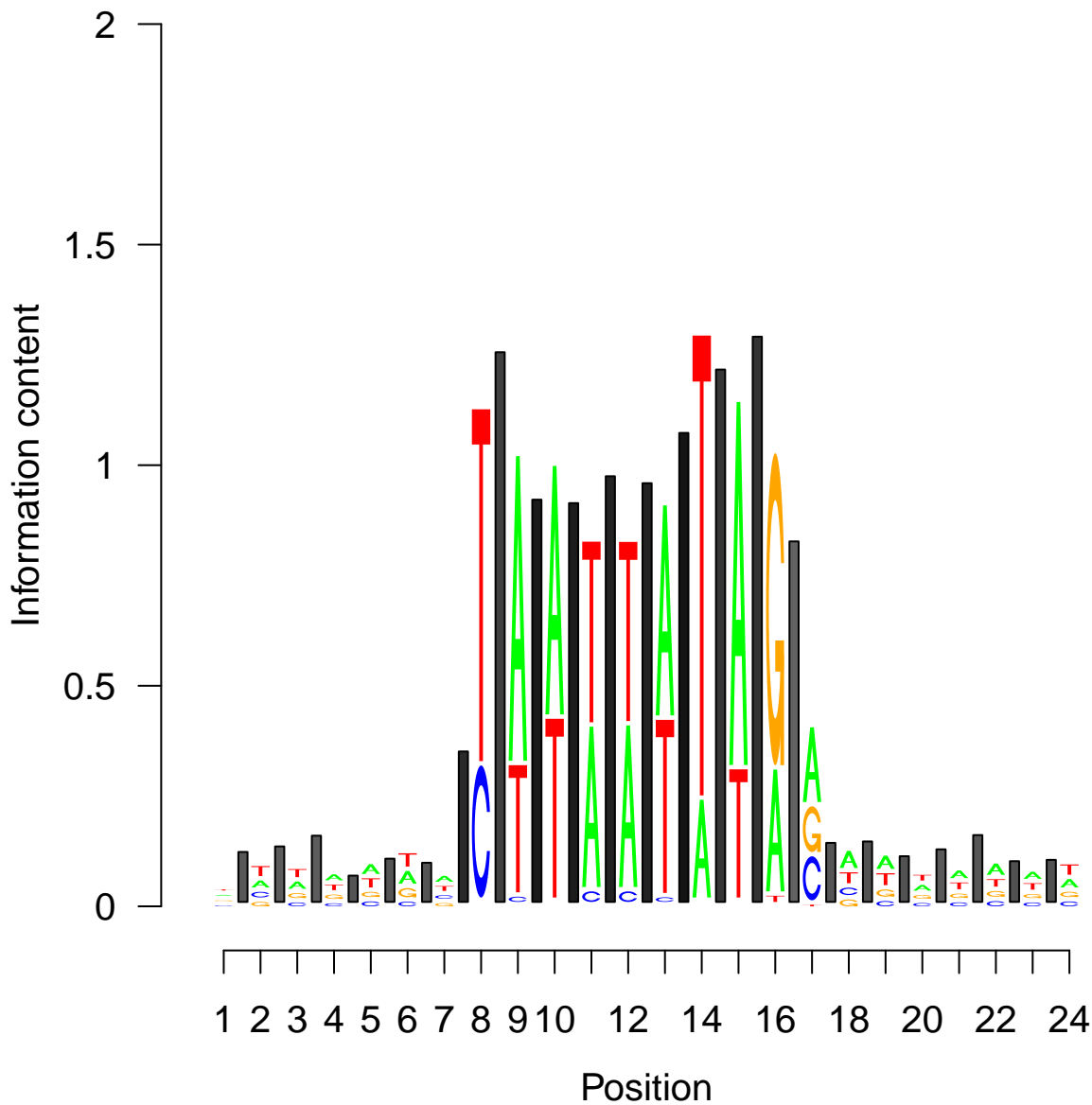

Supplement: Additional file 3: — TRX logo plots for general signatures of 16 transcription factor binding sites assembled from 416 Yeastract consensus sequences and collated according to JASPAR classification. (ZIP 1722 kb) [file 13029_2015_40_MOESM3_ESM.zip › SuppFileC/MADS.txt.txt.pdf]

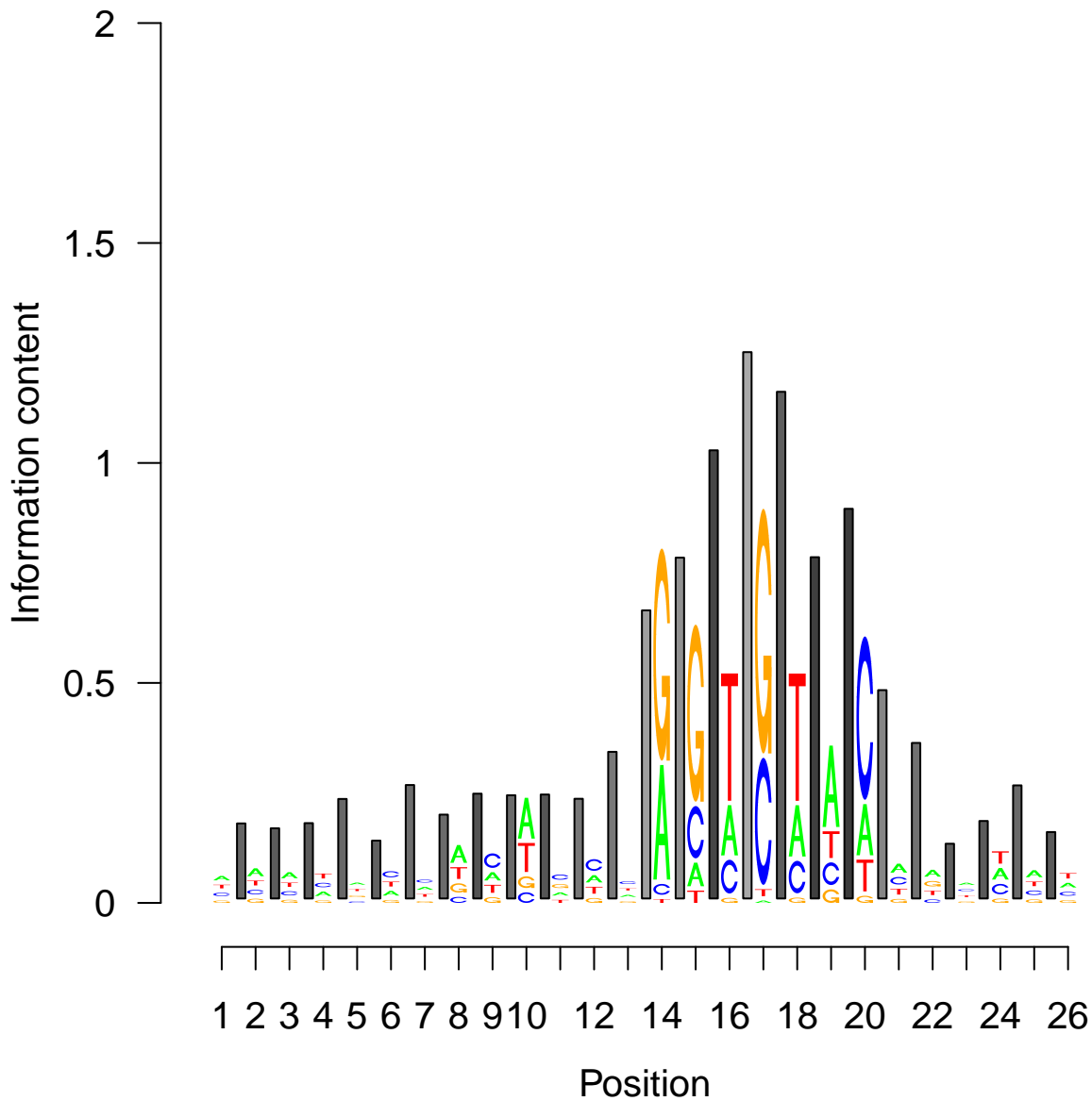

Supplement: Additional file 3: — TRX logo plots for general signatures of 16 transcription factor binding sites assembled from 416 Yeastract consensus sequences and collated according to JASPAR classification. (ZIP 1722 kb) [file 13029_2015_40_MOESM3_ESM.zip › SuppFileC/Myb.txt.txt.pdf]

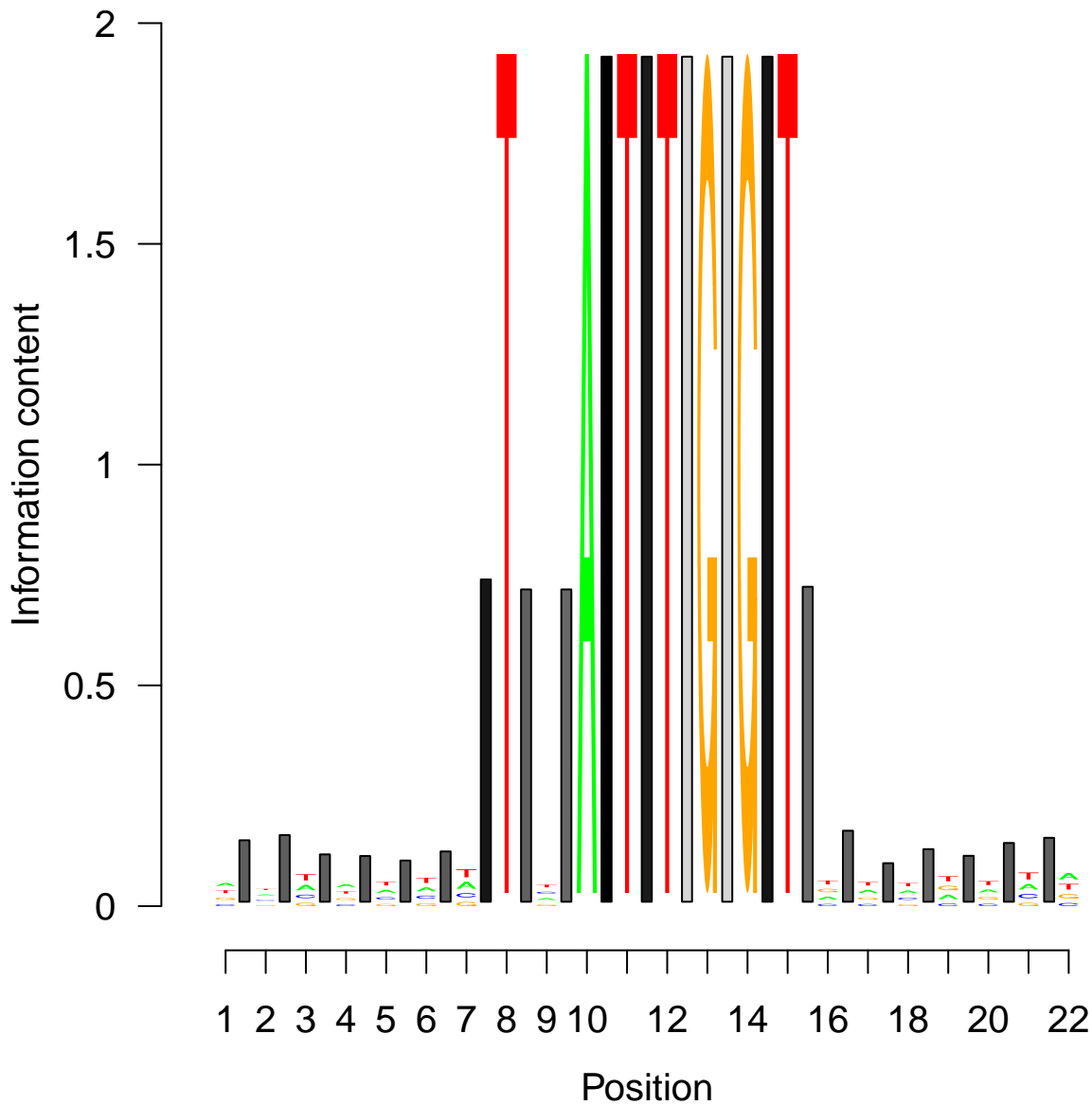

Supplement: Additional file 3: — TRX logo plots for general signatures of 16 transcription factor binding sites assembled from 416 Yeastract consensus sequences and collated according to JASPAR classification. (ZIP 1722 kb) [file 13029_2015_40_MOESM3_ESM.zip › SuppFileC/NFY_CCAAT-binding.txt.txt.pdf]

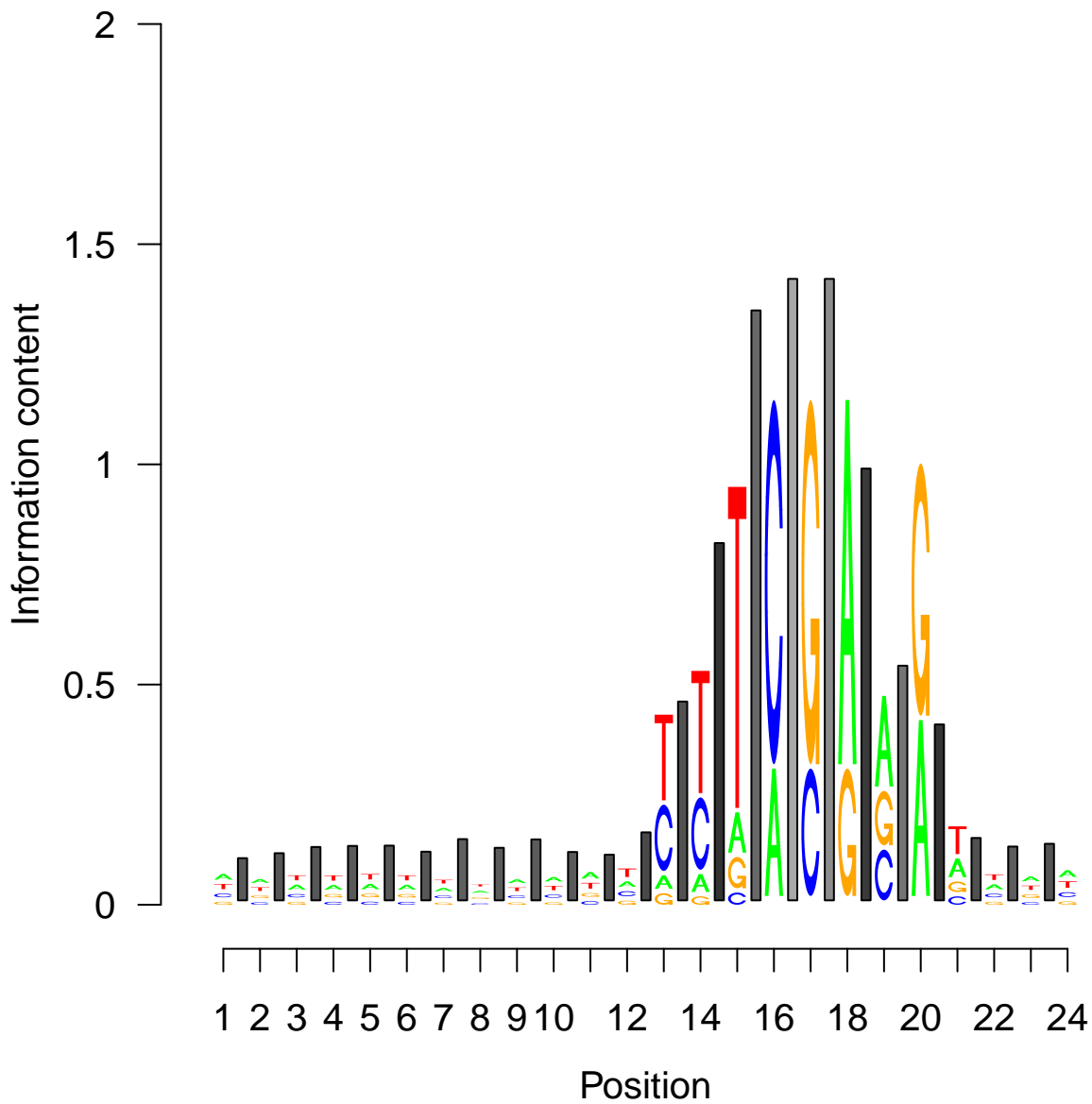

Supplement: Additional file 3: — TRX logo plots for general signatures of 16 transcription factor binding sites assembled from 416 Yeastract consensus sequences and collated according to JASPAR classification. (ZIP 1722 kb) [file 13029_2015_40_MOESM3_ESM.zip › SuppFileC/Rel.txt.txt.pdf]

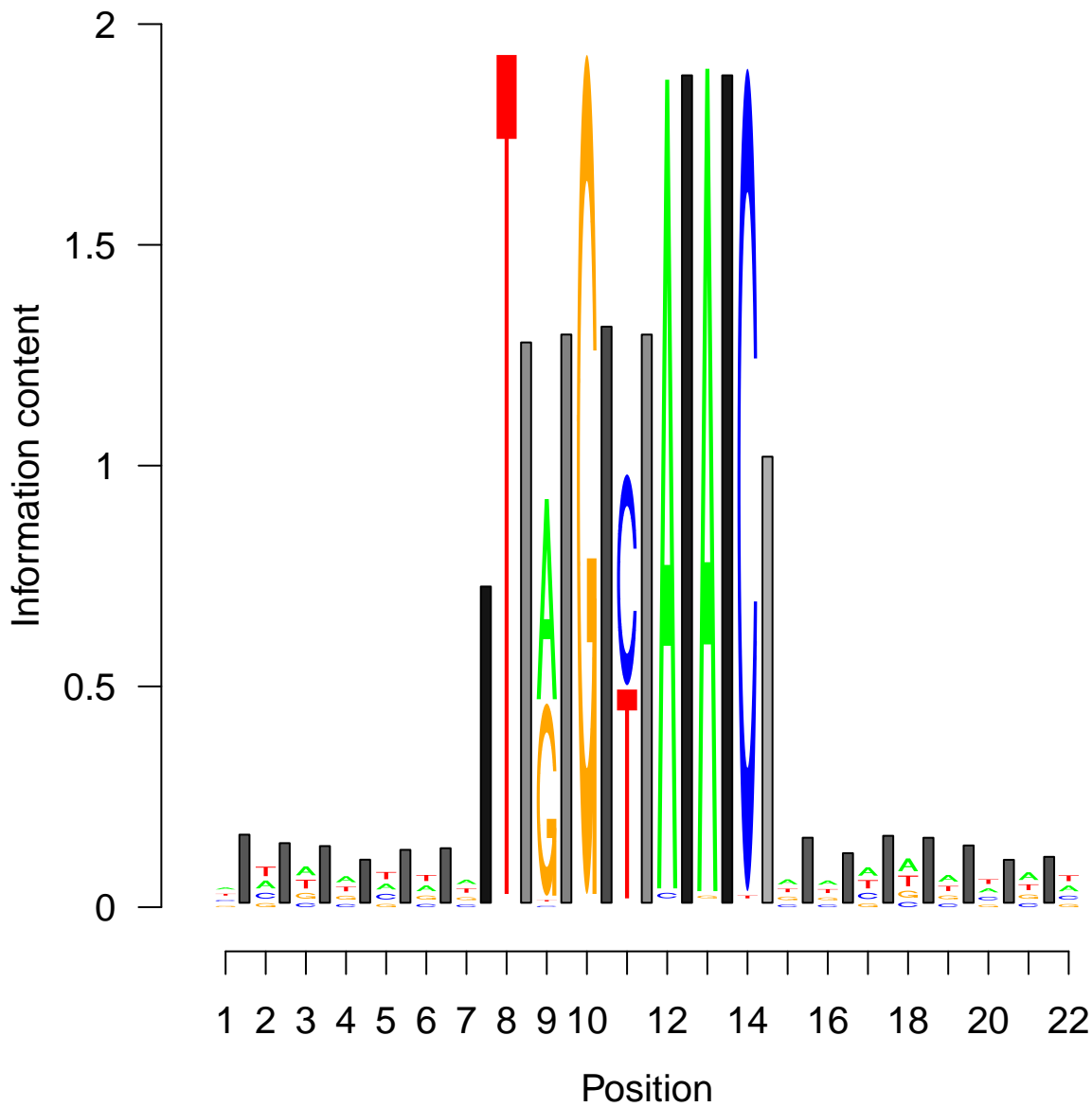

Supplement: Additional file 3: — TRX logo plots for general signatures of 16 transcription factor binding sites assembled from 416 Yeastract consensus sequences and collated according to JASPAR classification. (ZIP 1722 kb) [file 13029_2015_40_MOESM3_ESM.zip › SuppFileC/RFX.txt.txt.pdf]
